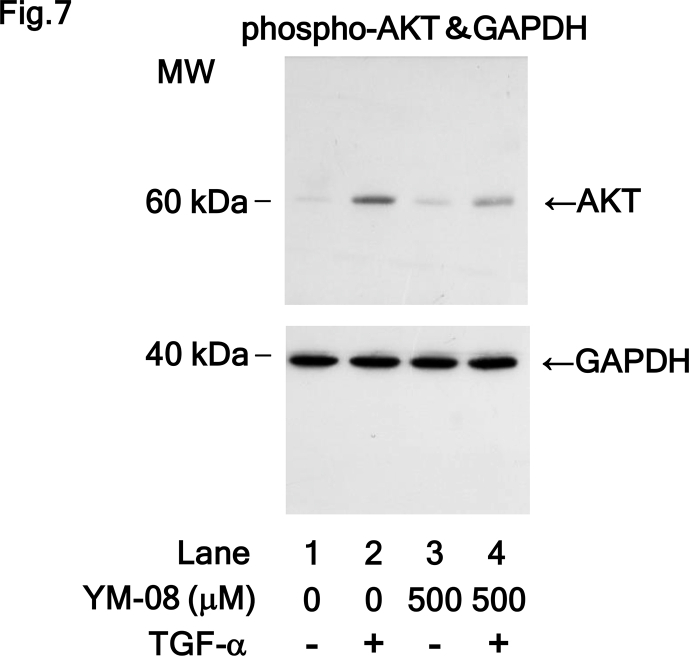# Heat shock protein 70 positively regulates transforming growth factor-α-induced hepatocellular carcinoma cell migration via the AKT signaling pathway

**DOI:** 10.1016/j.heliyon.2020.e05002

**Published:** 2020-09-23

**Authors:** Kaido Kobayashi, Rie Matsushima-Nishiwaki, Noriko Yamada, Saori Migita, Tomoyuki Hioki, Daisuke Mizutani, Osamu Kozawa

**Affiliations:** a Department of Pharmacology, Gifu University Graduate School of Medicine, Gifu, Japan; b Department of Dermatology, Kizawa Memorial Hospital, Minokamo, Gifu, Japan

**Keywords:** Cell biology, Biochemistry, Cancer research, Oncology, Laboratory medicine, AKT, Cell migration, HCC, HSP70, TGF-α

## Abstract

Heat shock proteins (HSPs) are induced in response to extracellular stress and manage the quality of proteins as molecular chaperones. HSP70, a highly conserved HSP, has been reported to correlate with the proliferation and migration of human cancer cells, such as oral, prostate, lung and liver cancer. Regarding hepatocellular carcinoma (HCC), the HSP70 levels in the tumor tissues from patients are significantly higher than those in the normal liver tissues. HSP70 reportedly upregulates the migration and invasion of HCC. The AKT, p38 mitogen-activated protein kinase (MAPK), c-*jun* N-terminal kinase (JNK) and Rho-kinase signaling pathways regulate the transforming growth factor (TGF)-α-induced migration of human HCC-derived HuH7 cells. However, the exact mechanism underlying the role of HSP70 in growth factor-induced HCC migration remains unclear. Therefore, in the present study, the mechanism underlying the involvement of HSP70 in TGF-α-induced HCC cell migration was investigated. Treatment with the HSP70 inhibitors VER155008 and YM-08 and the downregulation of HSP70 protein were confirmed to significantly suppress the TGF-α-induced cell migration of HuH7 cells. Both VER155008 and YM-08 reduced the TGF-α-induced phosphorylation of AKT without affecting the phosphorylation of p38 MAPK, JNK or Rho-kinase. These results strongly suggest that HSP70 positively regulates the TGF-α-induced migration of HCC cells via the AKT signaling pathway.

## 1 Introduction

Heat shock proteins (HSPs) are a family of highly conserved proteins, and their expression is controlled to low levels in cells under normal conditions [1, 2, 3]. However, in response to environmental stresses, such as heat shock, hypoxia and nutrient starvation, the protein expression of HSPs is upregulated, and these molecules play a cytoprotective role as molecular chaperones by stabilizing correct protein folding and suppressing protein aggregation [1, 2, 3].

HSPs are currently classified based on their molecular weight into the families of small HSP, HSP40, HSP60, HSP70, HSP90 and HSP110 [1, 3]. The small HSP family exert their function as chaperones in an ATP-independent manner, while other HSP families are ATP-dependent chaperones with ATPase activity [1, 3]. Since many oncoproteins require the high expression of HSP to maintain their function, the protein levels of HSPs are significantly higher in a wide range of cancer cells than in normal cells [3]. HSPs play a pivotal role in the progression of tumors and resistance against anti-cancer treatment and are currently considered therapeutic targets [1, 2, 3].

Among HSPs, HSP70 is reportedly correlated with the suppression of cell apoptosis and the promotion of cell proliferation and cell migration, leading to tumor progression [1, 2, 3, 4, 5, 6, 7, 8, 9]. In hepatocellular carcinoma (HCC), it has been shown that the expression of HSP70 is increased [2, 5]. Furthermore, its expression is markedly increased in HCC tumors of patients with portal vein invasion or lymph node metastasis [5], and the downregulation of HSP70 suppress HCC cells migration [8]. However, the detailed mechanisms underlying the role of HSP70 in HCC has not yet been fully elucidated.

Liver cancer is the fourth leading cause of cancer death worldwide [10]. The incidence of liver cancer is more than twice as high in men as in women, and liver cancer is the second leading cause of cancer death among men [10, 11]. Among primary liver cancer, the most common type (85%–90%) is HCC [10, 11]. The major risk factors for HCC development are chronic infection of hepatitis B virus (HBV) or hepatitis C virus (HCV) [10, 11, 12]. Other risk factors include alcohol abuse, exposure to aflatoxin B1, and fatty liver diseases caused by metabolic syndrome due to diabetes and obesity [10, 11, 12]. It is well known that intra- and extra-hepatic metastases and recurrence are very common in HCC [11]. Therefore, the prognosis after treatment for HCC, such as surgery, is poor [11, 12].

Dysregulation of growth factor/growth factor receptor signaling pathways is involved in HCC progression, including metastasis [13, 14, 15, 16]. The expression of transforming growth factor-alpha (TGF-α), a ligand of epidermal growth factor receptor (EGFR), has been known to be increased in HCC, including recurrent HCC after resection [13, 16]. Activation of the EGFR signaling pathway is known to promote the presence of HCC tumor cells in circulation and HCC cell metastasis [16]. In addition, TGF-α has been reported to the induce activation of the AKT, p38 mitogen activated protein kinase (MAPK), c-*jun* N-terminal kinase (JNK) and Rho-kinase signaling pathways as well as the subsequent migration of human HCC-derived HuH7 cells [17, 18, 19].

Although both HSP70 and growth factors are involved in HCC cell metastasis, few studies have examined the role of HSP70 in TGF-α-induced HCC cell migration. Therefore, the present study investigated the effect of HSP70 on TGF-α-induced HCC cell migration and clarified the underlying mechanism. Our findings showed that HSP70 positively regulated TGF-α-induced HCC cell migration via the AKT signaling pathway.

## 2 Materials and methods

### 2.1 Chemicals and antibodies

Recombinant human TGF-α was purchased from R&D Systems, Inc. (Minneapolis, MN). VER155008 and YM-08 were obtained from Sigma-Aldrich Co., Ltd. (St. Louis, MO). Control small interfering RNA (siRNA) (Silencer® Negative Control No.1 siRNA) and human HSP70-siRNA (sc-29352) were obtained from Life Technologies, Co. (Carlsbad, CA) and Santa Cruz Biotechnology, Inc. (Dallas, TX), respectively. Phospho-specific AKT (T308) antibodies (#13038), phospho-specific p38 MAPK antibodies (#4511), phospho-specific stress-activated protein kinase/JNK (JNK) antibodies (#4668), phospho-specific myosin phosphatase targeting subunit 1 (MYPT-1) antibodies (#4563) and phospho-specific p44/p42 MAPK antibodies (#9101) were purchased from Cell Signaling Technology, Inc. (Danvers, MA). Glyceraldehyde 3-phosphate dehydrogenase (GAPDH) antibodies (sc-47724) were obtained from Santa Cruz Biotechnology, Inc. An ECL Western blotting detection system and paraformaldehyde were purchased from GE Healthcare Life Sciences (Buckinghamshire, UK) and Alfa Aesar, Thermo Fisher Scientific Co. (Lancashire, UK), respectively. Other materials and chemicals were obtained from commercial sources.

VER155008 and YM-08 were dissolved in dimethyl sulfoxide (DMSO). When the maximum concentration of DMSO in the VER155008 or YM-08 solution was over 0.1%, we added the same concentration of DMSO to the control. A DMSO concentration of >0.1% in this study did not affect the cell migration assay or Western blot analyses.

### 2.2 Cell culture

Human HCC-derived HuH7 cells were obtained from the Japanese Collection of Research Bioresources Cell Bank (JCRB0403) (Tokyo, Japan). HuH7 cells were maintained in RPMI-1640 medium (Sigma-Aldrich Co.) containing 10% fetal calf serum (FCS; Hyclone; GE Healthcare Life Sciences) at 37 °C in a humidified atmosphere of 5% CO₂ and 95% air. For Western blot analyses, the cells were seeded into 100-mm diameter dishes (6 × 10⁵ cells/dish) in RPMI-1640 medium containing 10% FCS. The medium was exchanged for serum-free RPMI-1640 medium after three days, and then the cells were used for experiments after 24 h. For the cell migration assay, the cells were seeded into 100-mm diameter dishes (4 × 10⁵ cells/dish) in RPMI-1640 medium containing 10% FCS for 4 days and then used for experiments.

### 2.3 siRNA transfection

To downregulate HSP70 in HuH7 cells, the cultured cells were seeded into 6-well plates (2 × 10⁵ cells/well) in RPMI-1640 medium containing 10% FCS for 24 h and then transfected with HSP70-siRNA or negative control-siRNA using siLentFect Lipid Reagent (Bio Rad Laboratories, Inc., Hercules, CA) according to the manufacturer's instructions. The cells were incubated for 24 h at 37 °C with siRNA (50 nM)-siLentFect complexes and then used for the cell migration assay.

### 2.4 Cell migration assay

A transwell cell migration assay using a Boyden chamber (polycarbonate membrane with 8-μm pores, Transwell; Corning Costar Co., Cambridge, MA) was performed as described previously [17, 18, 19, 20, 21]. The cells (1 × 10⁵ cells/well) were seeded onto the upper chamber in serum-free RPMI-1640 medium. The seeded cells were pretreated with VER155008 or YM-08 for 60 min at 37 °C in the upper chamber. TGF-α was then added to the lower chamber for 23 h at 37 °C. After incubation, the migrated cells attached to the underside of the membrane were fixed with 4% paraformaldehyde for 20 min at room temperature, and the cells on the upperside of the membrane were removed. The migrated cells were then stained with 4’,6-diamino-2-phenylindole (DAPI) solution for 10 min at room temperature. Images of the migrated cells were captured and counted using fluorescent microscopy at a magnification of 20× by counting the stained cells in 3 randomly chosen high-power fields.

### 2.5 Western blot analyses

To examine the effect of HSP70 on the TGF-α-stimulated AKT, p38 MAPK, JNK, Rho-kinase and p44/p42 MAPK signaling pathways, the cultured cells were pretreated with the indicated doses of VER155008 or YM-08, and then stimulated by TGF-α (30 ng/ml) for 1 min for AKT and MYPT-1, 5 min for p38 MAPK and p44/p42 MAPK, and 20 min for JNK. The cultured cells were washed twice with ice-cold phosphate-buffered saline (PBS). The cells were then lysed and sonicated in 800 μl lysis buffer (62.5 mM Tris-HCl [pH 6.8], 2% sodium dodecyl sulfate [SDS], 50 mM dithiothreitol and 10% glycerol). SDS-polyacrylamide gel electrophoresis (PAGE) was performed with the cell lysate as described previously [17, 18, 19, 20, 21]. Western blot analyses were performed using phospho-specific AKT (T308; 1:200000), phospho-specific p38 MAPK (1:20000), phospho-specific JNK (1:1000), phospho-specific MYPT-1 (1:1000), phospho-specific p44/p42 MAPK (1:50000) and GAPDH (1:1000) as primary antibodies. Horseradish peroxidase (HRP)-labeled anti-rabbit IgG (Seracare Life Sciences, Milford, MA) were used for secondary antibodies except for GAPDH antibodies, and HRP-labeled anti-mouse IgG antibodies (Cell Signaling Technology, Inc.) were used for GAPDH antibodies. The HRP activity on a polyvinylidene difluoride membrane (Bio-Rad Laboratories, Inc.) was visualized on an X-ray film using the ECL Western blotting detection system. Densitometric analyses were performed using a scanner and an image analysis software program (ImageJ v1.48; National Institutes of Health, Bethesda, MD). Each background-subtracted phosphorylation signal intensity was normalized to the respective GAPDH signal, and then the fold increase was plotted in comparison with that of the control cells without stimulation.

### 2.6 Statistical analyses

The data were analyzed using an analysis of variance (ANOVA) with Tukey's post hoc test to determine whether or not significant differences existed. *P* < 0.05 was considered statistically significant. The data are presented as the mean ± standard deviation (SD) from three independent cell culture experiments.

## 3 Results

### 3.1 Effects of VER155008 or YM-08 on the TGF-α-induced migration of HuH7 cells

TGF-α has been reported to induce the migration of human HCC-derived HuH7 cells in a transwell cell migration assay [17, 18, 19, 21]. In contrast, the knockdown of HSP70 suppresses migration and invasion of the HCC cells, SMMC7721 and Hep3B [8]. Therefore, we first examined whether or not VER155008, an inhibitor of HSP70 [22], affects the TGF-α-induced HuH7 cell migration.

**Figure 1:**
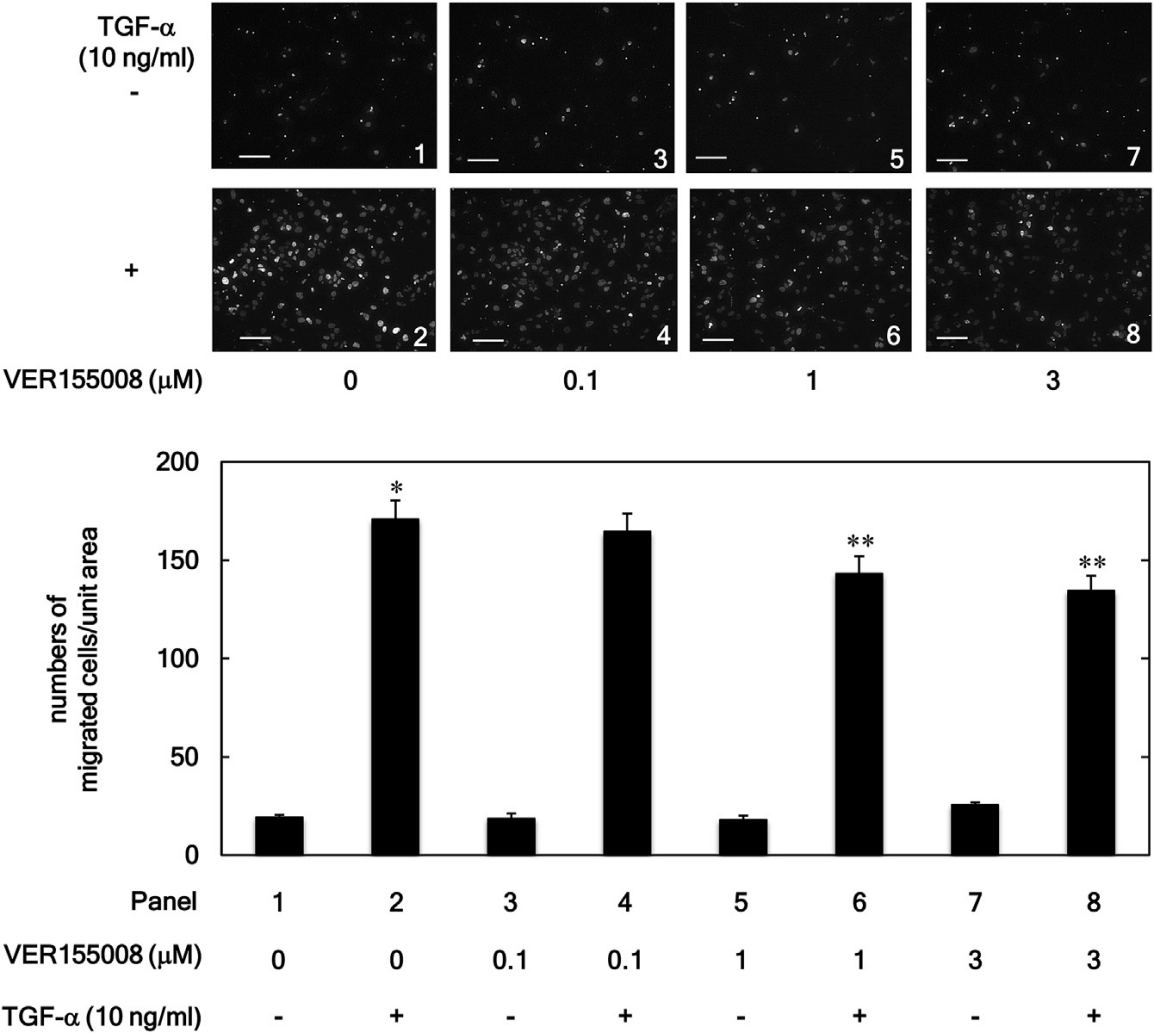
Effects of VER155008 on the TGF-α-induced HuH7 cell migration. The cells were pretreated with the indicated concentration of VER155008 for 60 min and then stimulated by TGF-α (10 ng/ml) or vehicle for 23 h. The migrated cells were stained with DAPI for the nucleus and then photographed by fluorescent microscopy at a magnification of 20× (upper panel) and counted (bar graph). Each value represents the mean ± SD of triplicate determinations. ∗*p* < 0.05, versus column 1; ∗∗*p* < 0.05, versus column 2. Scale bar: 100 μm.

**Figure 2:**
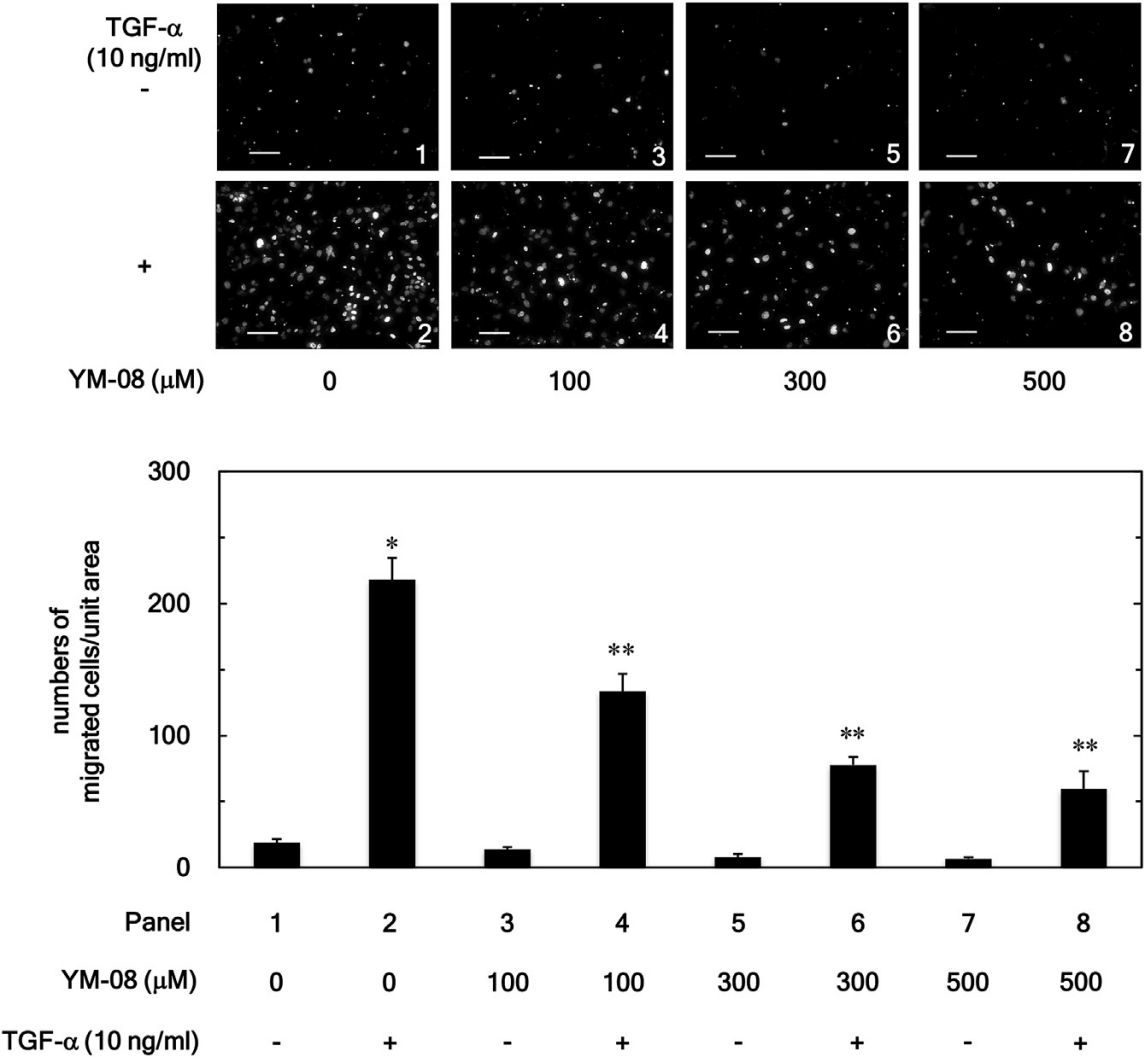
Effects of YM-08 on the TGF-α-induced HuH7 cell migration. The cells were pretreated with the indicated concentration of YM-08 for 60 min and then stimulated by TGF-α (10 ng/ml) or vehicle for 23 h. The migrated cells were stained with DAPI for the nucleus and then photographed by fluorescent microscopy at a magnification of 20× (upper panel) and counted (bar graph). Each value represents the mean ± SD of triplicate determinations. ∗*p* < 0.05, versus column 1; ∗∗*p* < 0.05, versus column 2. Scale bar: 100 μm.

As shown in Figure 1, VER155008 was confirmed to significantly suppress the migration of HuH7 cells induced by TGF-α (10 ng/ml) at concentrations from 1 to 3 μM. Furthermore, VER155008 alone did not affect the cell migration at these concentrations. In addition to VER155008, another type of HSP70 inhibitor, YM-08 [23], also significantly inhibited the TGF-α-induced migration of HuH7 cells in a dose-dependent manner over the range 100–500 μM (Figure 2).

### 3.2 Effects of HSP70-siRNA on the TGF-α-induced migration of HuH7 cells

**Figure 3:**
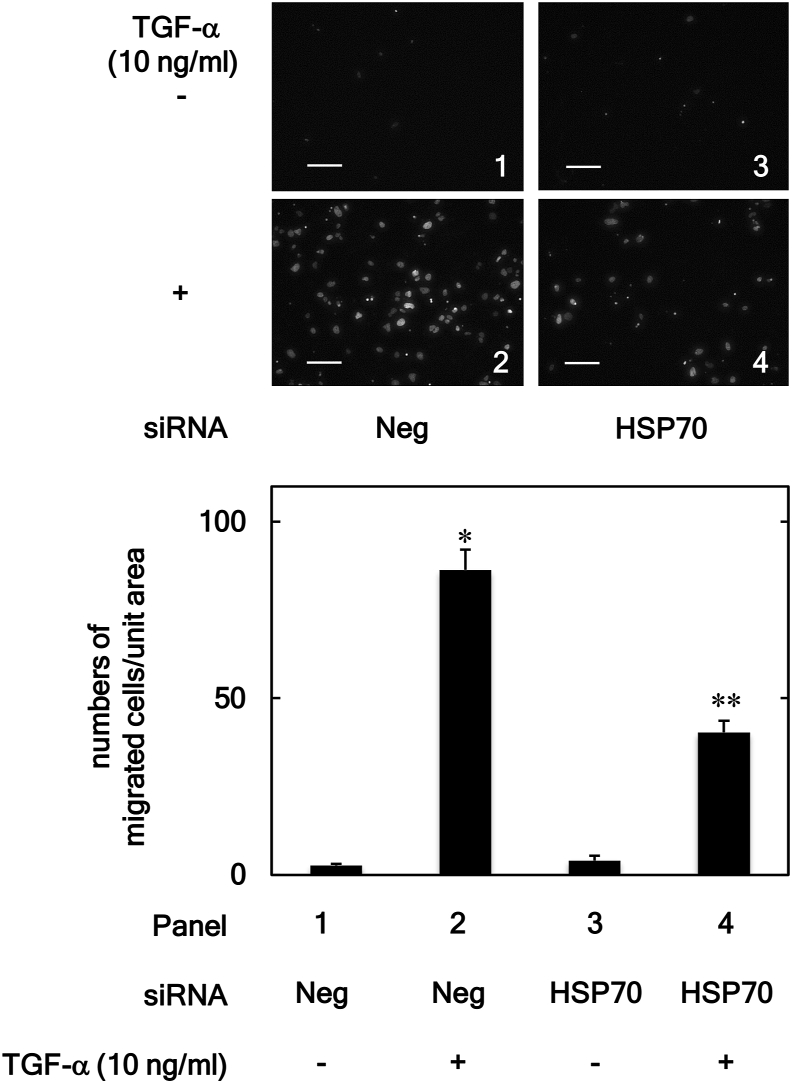
Effects of HSP70-siRNA on the TGF-α-induced HuH7 cell migration. The HSP70-siRNA (HSP70) or negative control-siRNA (Neg) transfected cells were stimulated by TGF-α (10 ng/ml) or vehicle for 24 h. The migrated cells were stained with DAPI for the nucleus and then photographed by fluorescent microscopy at a magnification of 20× (upper panel) and counted (bar graph). Each value represents the mean ± SD of triplicate determinations. ∗*p* < 0.05, versus column 1; ∗∗*p* < 0.05, versus column 2. Scale bar: 100 μm.

To further confirm the effect of HSP70 on the TGF-α-induced HuH7 cell migration, the effect of HSP70 knockdown on the TGF-α-induced migration of HuH7 cells was examined using siRNA targeting HSP70. As shown in Figure 3, compared to the negative control-siRNA transfection, HSP70-siRNA transfection significantly suppressed the TGF-α (10 ng/ml)-induced migration of HuH7 cells.

### 3.3 Effects of VER155008 on TGF-α-induced phosphorylation of p38 MAPK, JNK, MYPT-1 or p44/p42 MAPK in HuH7 cells

P38 MAPK, JNK and Rho-kinase signaling pathways function as positive regulators in TGF-α induced HuH7 cell migration [17, 19]. However, the activation of p44/p42 MAPK is closely associated with HCC development and HuH7 cell proliferation [24, 25] but is not involved in the TGF-α-induced migration of HuH7 cells [19]. Therefore, the effects of VER155008 on TGF-α-stimulated phosphorylation of p38 MAPK, JNK, Rho-kinase substrate MYPT-1 and p44/p42 MAPK in HuH7 cells were measured.

**Figure 4:**
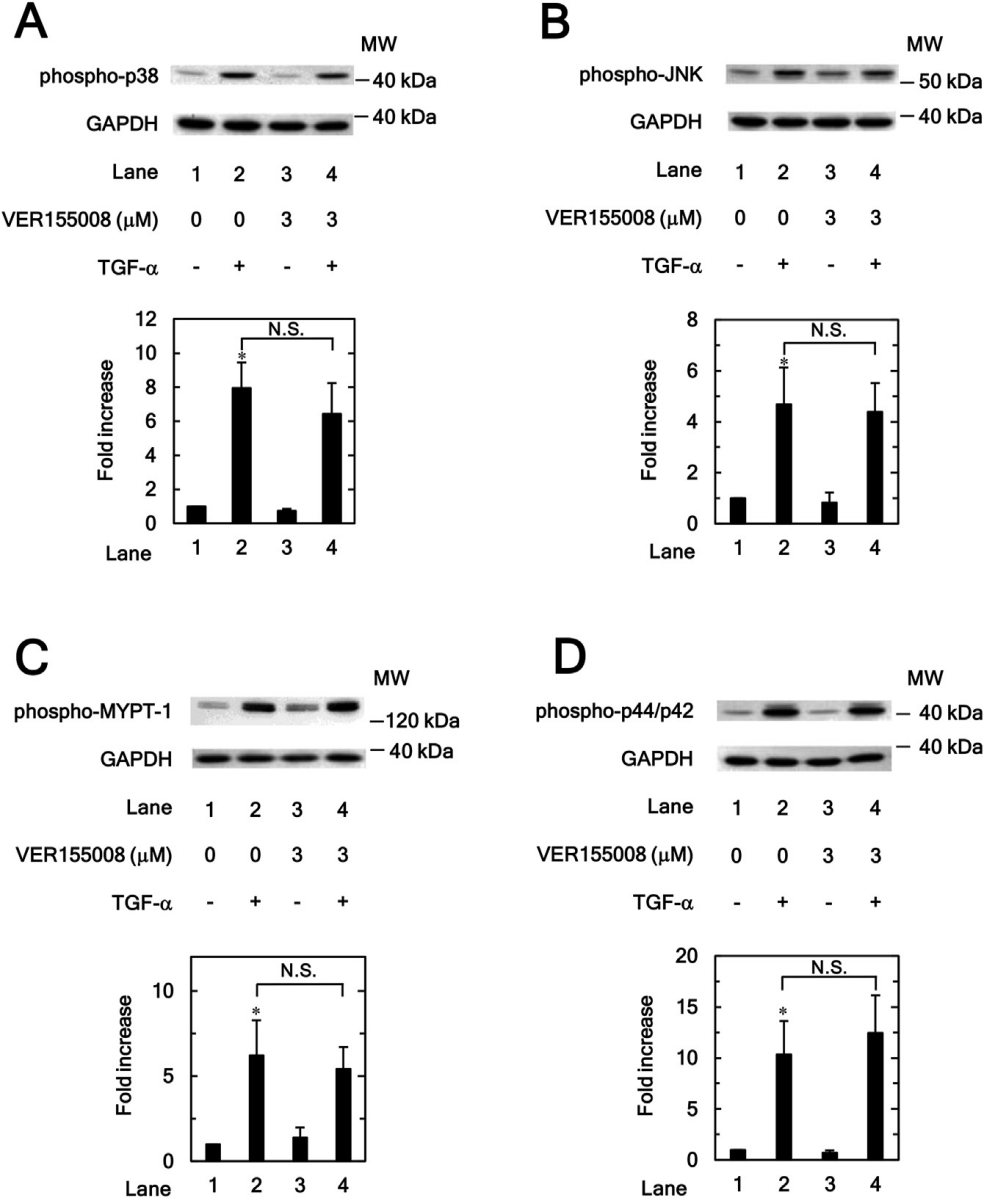
Effects of VER155008 on the TGF-α-induced phosphorylation of p38 MAPK (A), 54 kDa JNK (B), MYPT-1 (C) and p44/p42 MAPK (D) in HuH7 cells. The cells were pretreated with VER155008 (3 μM) or vehicle for 60 min, and then stimulated by TGF-α (30 ng/ml) or vehicle for 5 min for p38 NAPK, 20 min for JNK, 1 min for MYPT-1 and 5min for p44/p42 MAPK. Western blot analyses using antibodies against phospho-specific p38 MAPK, phospho-specific JNK, phospho-specific MYPT-1, phospho-specific p44/p42 MAPK and GAPDH were performed. The bar graphs represent the relative levels of p38 MAPK (A), 54 kDa JNK (B), MYPT-1 (C) and p44/p42 MAPK (D) phosphorylation. The phosphorylation levels were corrected by the levels of GAPDH and then expressed as the fold increase compared with the basal levels presented in lane 1. Each value represents the mean ± SD of triplicate determinations from three independent cell preparations. ∗*p* < 0.05, versus lane 1. N.S. = Not significant. Non-adjusted images of Figures 4A, 4B, 4C and 4D are provided as supplementary materials (Figure S1).

The TGF-α-stimulated phosphorylation of p38 MAPK (Figure 4A), JNK (Figure 4B) and MYPT-1 (Figure 4C) was not significantly suppressed by VER155008. In addition, VER155008 also showed no inhibition of TGF-α-induced p44/p42 MAPK phosphorylation (Figure 4D).

### 3.4 Effect of VER155008 on TGF-α-induced phosphorylation of AKT in HuH7 cells

**Figure 5:**
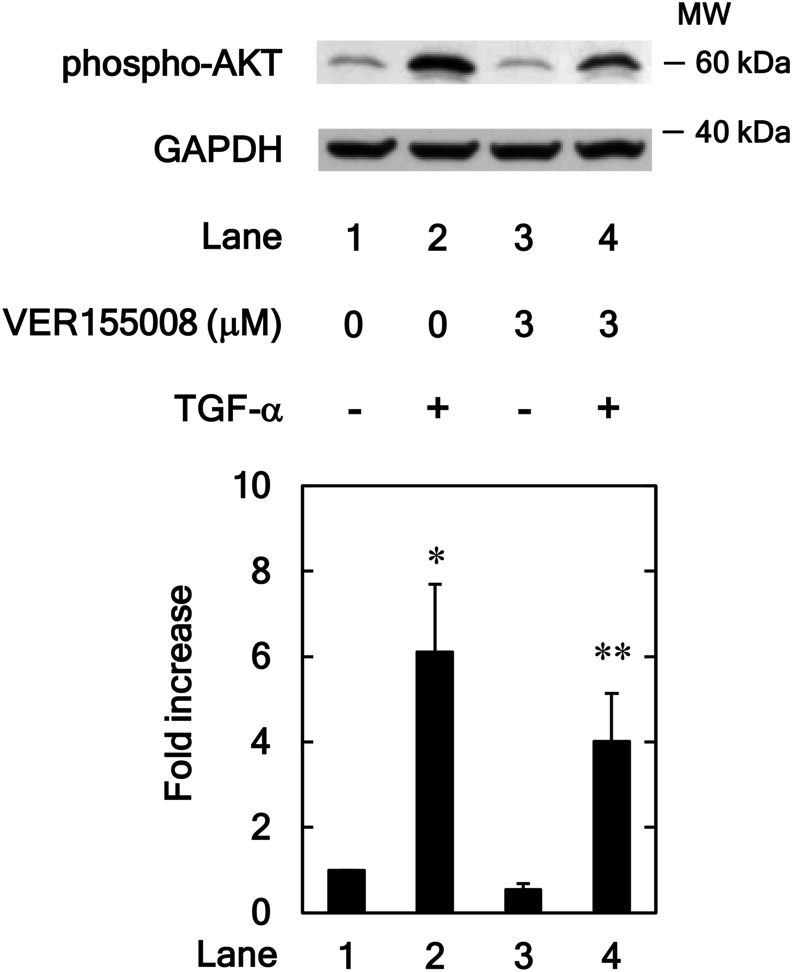
Effects of VER155008 on the TGF-α-induced phosphorylation of AKT in HuH7 cells. The cells were pretreated with VER155008 (3 μM) or vehicle for 60 min and then stimulated by TGF-α (30 ng/ml) or vehicle for 1 min. Western blot analyses using antibodies against phospho-specific AKT and GAPDH were performed. The bar graph represents the relative levels of AKT phosphorylation. The phosphorylation levels were corrected by the levels of GAPDH and then expressed as the fold increase compared with the basal level presented in lane 1. Each value represents the mean ± SD of triplicate determinations from three independent cell preparations. ∗*p* < 0.05, versus lane 1; ∗∗*p* < 0.05, versus lane 2. Non-adjusted images of Figure 5 are provided as supplementary materials (Figure S2).

In addition to the p38 MAPK, JNK and Rho-kinase signaling pathways, the TGF-α induced migration of HuH7 cells is also mediated by the AKT signaling pathway [18]. Therefore, to investigate whether or not HSP70 affects the TGF-α induced HuH7 cell migration via the AKT pathway, the effect of VER155008 was examined on the TGF-α-stimulated phosphorylation of AKT. VER155008 significantly downregulated the TGF-α-stimulated phosphorylation of AKT (Figure 5) at a concentration of 3 μM.

### 3.5 Effects of YM-08 on TGF-α-induced phosphorylation of p38 MAPK, JNK, MYPT-1 or p44/p42 MAPK in HuH7 cells

**Figure 6:**
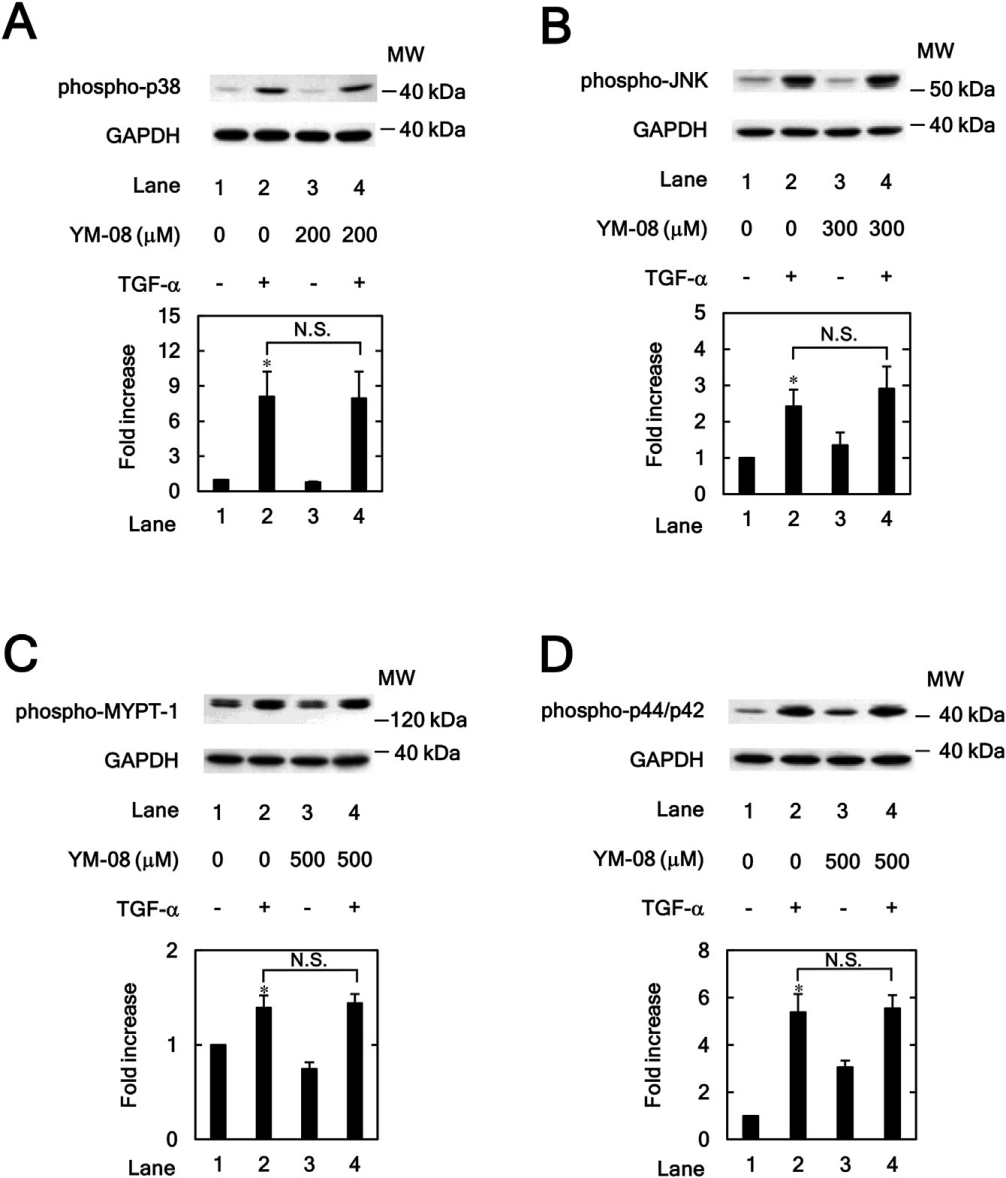
Effects of YM-08 on the TGF-α-induced phosphorylation of p38 MAPK (A), 54 kDa JNK (B), MYPT-1 (C) and p44/p42 MAPK (D) in HuH7 cells. The cells were pretreated with the indicated concentration of YM-08 for 60 min for p38 MAPK and JNK and 2 h for MYPT-1 and p44/p42 MAPK and then stimulated by TGF-α (30 ng/ml) or vehicle for 5 min for p38 MAPK, 20 min for JNK, 1 min for MYPT-1 and 5 min for p44/42 MAPK. Western blot analyses using antibodies against phospho-specific p38 MAPK, phospho-specific JNK, phospho-specific MYPT-1, phospho-specific p44/p42 MAPK and GAPDH were performed. The bar graphs represent the relative levels of p38 MAPK (A), 54 kDa JNK (B), MYPT-1 (C) and p44/p42 MAPK (D) phosphorylation. The phosphorylation levels were corrected by the levels of GAPDH and then expressed as the fold increase compared with the basal levels presented in lane 1. Each value represents the mean ± SD of triplicate determinations from three independent cell preparations. ∗*p* < 0.05, versus lane 1. N.S. = Not significant. Non-adjusted images of Figures 6A, 6B, 6C and 6D are provided as supplementary materials (Figure S3).

In addition to VER155008, the effect of YM-08 on the TGF-α-stimulated phosphorylation of p38 MAPK, JNK, MYPT-1 and p44/p42 MAPK was examined. The TGF-α-stimulated phosphorylation of p38 MAPK and JNK was not inhibited by YM-08 at concentrations of 200 μM (Figure 6A) and 300 μM (Figure 6B), respectively. The phosphorylation of p38 MAPK and JNK was upregulated by treatment with >200 μM of YM-08 alone for p38 MAPK and >300 μM of YM-08 alone for JNK, presumably because p38 MAPK and JNK are stress-activated protein kinases [26]. Thus, it was not possible to examine the effect of YM-08 above these concentrations on TGF-α-induced p38 MAPK and JNK activities. In addition, the TGF-α-stimulated phosphorylation of MYPT-1 (Figure 6C) and p44/p42 MAPK (Figure 6D) was not affected by YM-08 (500 μM).

### 3.6 Effect of YM-08 on TGF-α-induced phosphorylation of AKT in HuH7 cells

**Figure 7:**
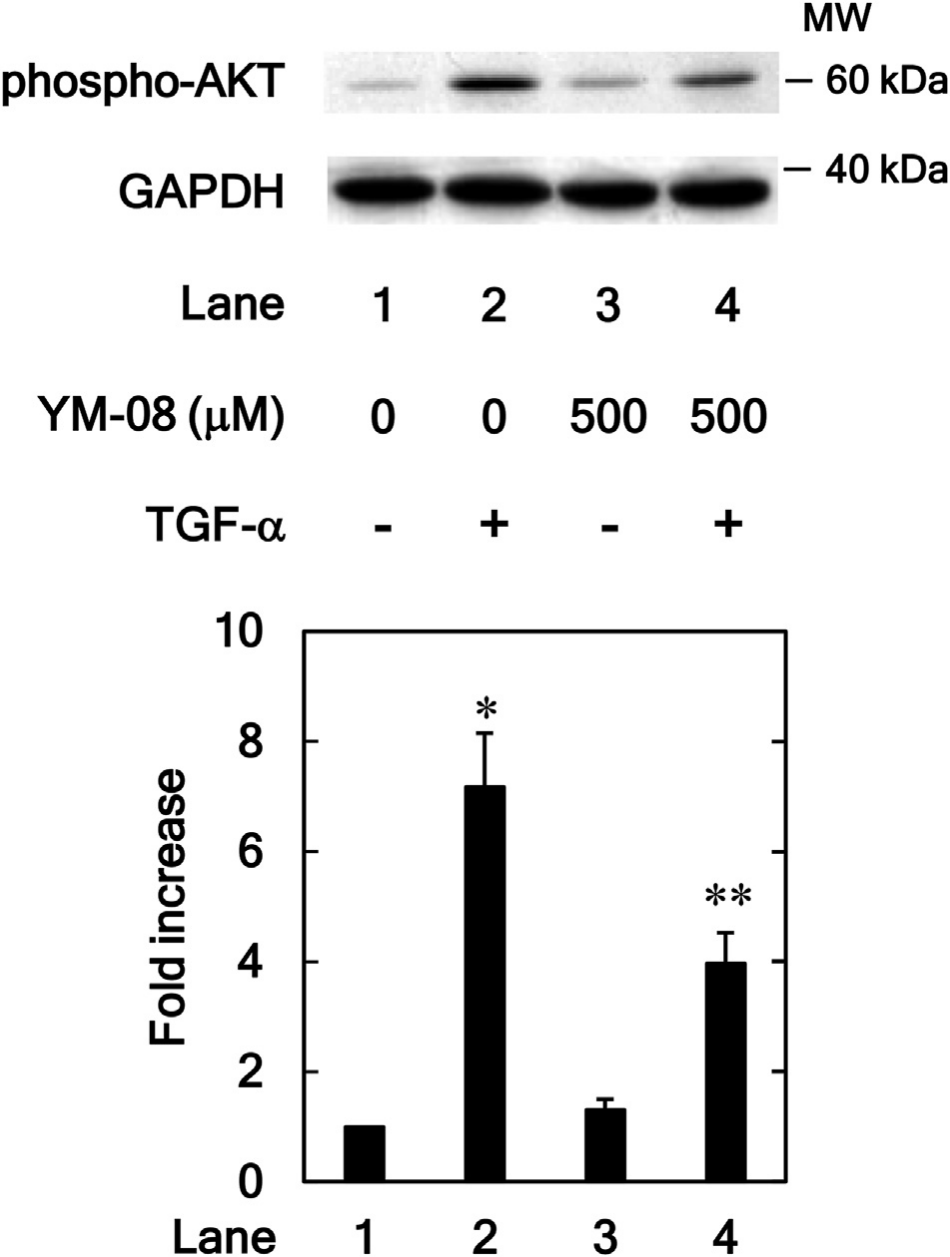
Effects of YM-08 on the TGF-α-induced phosphorylation of AKT in HuH7 cells. The cells were pretreated with YM-08 (500 μM) or vehicle for 2h and then stimulated by TGF-α (30 ng/ml) or vehicle for 1 min. Western blot analyses using antibodies against phospho-specific AKT and GAPDH were performed. The bar graphs represent the relative levels of AKT phosphorylation. The phosphorylation levels were corrected by the levels of GAPDH and then expressed as the fold increase compared with the basal levels presented in lane 1. Each value represents the mean ± SD of triplicate determinations from three independent cell preparations. ∗*p* < 0.05, versus lane 1; ∗∗*p* < 0.05, versus lane 2. Non-adjusted images of Figure 7 are provided as supplementary materials (Figure S4).

The effect of YM-08 was also examined on the TGF-α-stimulated phosphorylation of AKT. As shown in Figure 7, YM-08 (500 μM) significantly suppressed the TGF-α-stimulated phosphorylation of AKT as VER155008. Treatment with YM-08 alone did not affect the activity of AKT in HuH7 cells.

## 4 Discussion

In HCC cells and tumor tissues from HCC patients, it is generally recognized that the HSP70 expression is elevated [2, 5]. The high expression of HSP70 is associated with lymph node metastasis and portal vein invasion of HCC [2, 5]. Furthermore, it has been reported that HCC cell migration is induced by the upregulation of HSP70 expression [6], and HSP70 knockdown inhibits it [8]. These reports indicate that HSP70 positively regulates the migration of HCC cells. However, the exact mechanism by which HSP70 regulates the migration of HCC cells has been unclear. The TGF-α/EGFR signaling pathway is well known to be involved in HCC development, including HCC cell migration [13, 16]. Previously, TGF-α was reported to induce the migration and invasion of HCC-derived HuH7 cells [17, 18, 19, 21]. The TGF-α-induced HuH7 cell migration is mediated through the AKT, p38 MAPK, JNK and Rho-kinase signaling pathways [17, 18, 19]. Although both HSP70 and TGF-α/EGFR signaling pathway are presumed to be involved in HCC cell migration, there have been few reports on their relationship in HCC cell migration. Therefore, we investigated the mechanism of action underlying the role of HSP70 in TGF-α-induced migration of HuH7 cells.

In the present study, the HSP70 inhibitors VER155008 and YM-08 and the downregulation of HSP70 using siRNA were found to significantly suppress the TGF-α-induced migration of HuH7 cells. The HSP70 inhibitors suppressed the TGF-α-stimulated phosphorylation of AKT in HuH7 cells, although they failed to affect the TGF-α-induced activation of p38 MAPK, JNK, Rho-kinase or p44/p42 MAPK. HSP70 reportedly binds to JNK or p38 MAPK and blocks their activation [1, 3, 27]. Furthermore, HSP70 inhibits senescence by suppressing the phosphoinositide 3-kinase (PI3K)/AKT and Ras-p44/p42 MAPK signaling pathways in multiple types of cancer cells [3]. However, HSP70 likely positively regulates the signaling pathway of AKT but not those of p38 MAPK, JNK or Rho-kinase in TGF-α-induced HCC cell migration.

**Figure 8:**
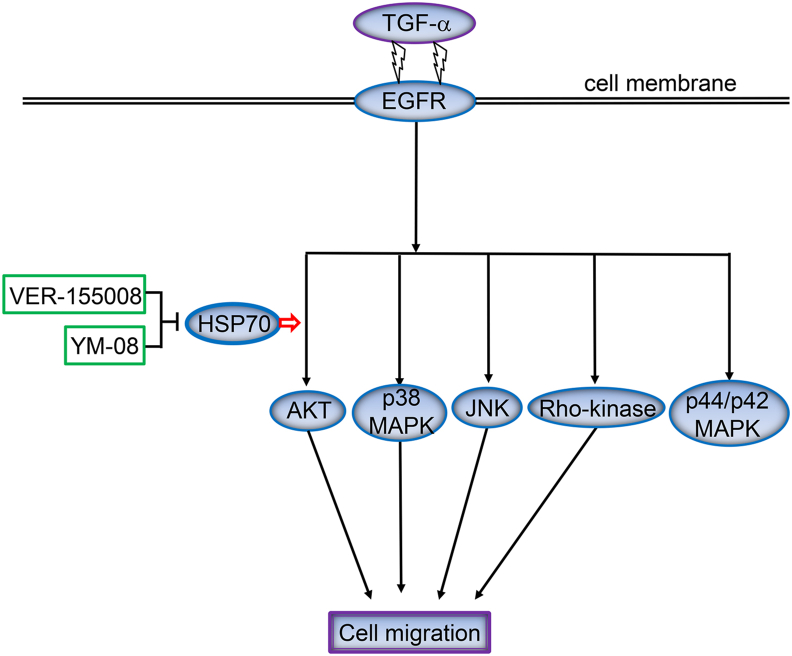
A schematic illustration of the regulatory mechanism underlying the role of HSP70 in TGF-α-induced HCC cell migration.

HSP70 has been shown to serve as a co-chaperone to HSP90 and maturates and stabilizes many oncogenic HSP90 client kinases, including AKT [3]. In addition, the PI3K/AKT signaling pathway has been reported to be activated by the interaction of HSP70 with growth factors, such as nerve growth factor and platelet-derived growth factor [1]. Co-chaperoning activities of HSP70 to HSP90 or some interaction between HSP70 and TGF-α may be involved in the positive regulatory activity of HSP70 on the TGF-α-induced activation of AKT in HCC cells. The effect of HSP70 on cell signaling pathways may be influenced by the type of growth factor that stimulates the signaling pathways or may depend on the cell type and environment. The potential role of HSP70 in the regulation of TGF-α-induced migration of HCC cells shown in this study is summarized in Figure 8.

Epithelial-to-mesenchymal transition (EMT) is known to play an important role in the invasion and metastasis of cancers including HCC [28, 29]. Tumor cells acquire the ability to migrate via EMT [28, 29]. Activation of the PI3K/AKT, p38 MAPK, JNK and p44/p42 MAPK signaling pathways is reportedly involved in the EMT of HCC [5, 28, 29]. Extracellular HSP70 induces EMT via the p38 MAPK signaling pathway in HCC cells [5], whereas EGF, a ligand of EGFR as TGF-α, induces EMT via the activation of the p44/p42 MAPK and AKT signaling pathways in HCC [28, 29]. In the present study, HSP70 promoted TGF-α-induced HCC cell migration through the AKT signaling pathway. The AKT signaling pathway stimulated by the combined effects of TGF-α and HSP70 may have induced EMT, which then caused HCC cell migration.

The targets of the AKT signaling pathway that play an intimate role in EMT, which is key for HCC cell migration, remain unclear. The upregulation of the expression of the transcription factors slug and snail and the downregulation of E-cadherin expression, which are induced by the activation of the AKT signaling pathway, have been reported to be involved in EMT induction in HCC [28, 29]. While, the stabilization of the high expression of β-catenin via the activation of the tumor necrosis factor-α-stimulated PI3K/AKT signaling pathway is also involved in the EMT of HCC cells [29]. Further studies will be needed to elucidate the targets involved in the induction of EMT downstream of the HSP70-accelerated TGF-α-activated AKT signaling pathway. Since HSP70 plays complicated roles in cancer, further investigations are needed to clarify the exact function of HSP70 in HCC development and recurrence.

Taken together, the present findings strongly suggest that HSP70 positively regulates the AKT signaling pathway in HCC cells, leading to the promotion of TGF-α-induced cell migration.

## Declarations

### Author contribution statement

R. Matsushima-Nishiwaki: Conceived and designed the experiments; Performed the experiments; Analyzed and interpreted the data; Wrote the paper.

O. Kozawa: Conceived and designed the experiments; Analyzed and interpreted the data; Wrote the paper.

K. Kobayashi: Performed the experiments; Analyzed and interpreted the data; Wrote the paper.

N. Yamada: Performed the experiments; Analyzed and interpreted the data.

S. Migita. T. Hioki and D. Mizutani: Performed the experiments.

### Funding statement

R. Matsushima-Nishiwaki was supported by 10.13039/501100001691Japan Society for the Promotion of Science, Ministry of Education, Culture, Sports, Science and Technology of Japan (JP17K09417). O. Kozawa was supported by 10.13039/501100001691Japan Society for the Promotion of Science, Ministry of Education, Culture, Sports, Science and Technology of Japan (JP25460989).

### Competing interest statement

The authors declare no conflict of interest.

### Additional information

Supplementary content related to this article has been published online at https://doi.org/10.1016/j.heliyon.2020.e05002.

## Figures and Tables

**S1:**
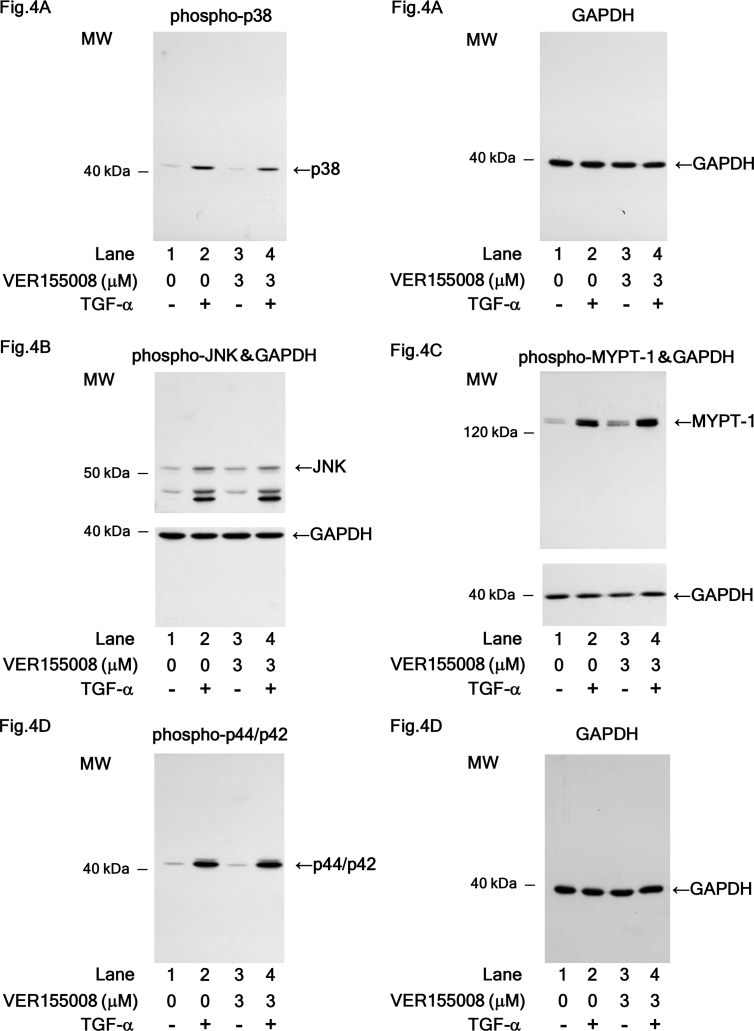
**Figures S1, S2 and S4.** For Figures 4B, 4C, 5 and 7, the blotted membranes have been cut just above 40 kDa according to protein markers in order to examine the phosphorylated proteins and GAPDH as a housekeeping protein on the same membrane. Therefore, the top (for the phosphorylated proteins) and the bottom (for GAPDH) of each membrane have been detected separately. Thus, the images of these figures are cropped.

**S2:**
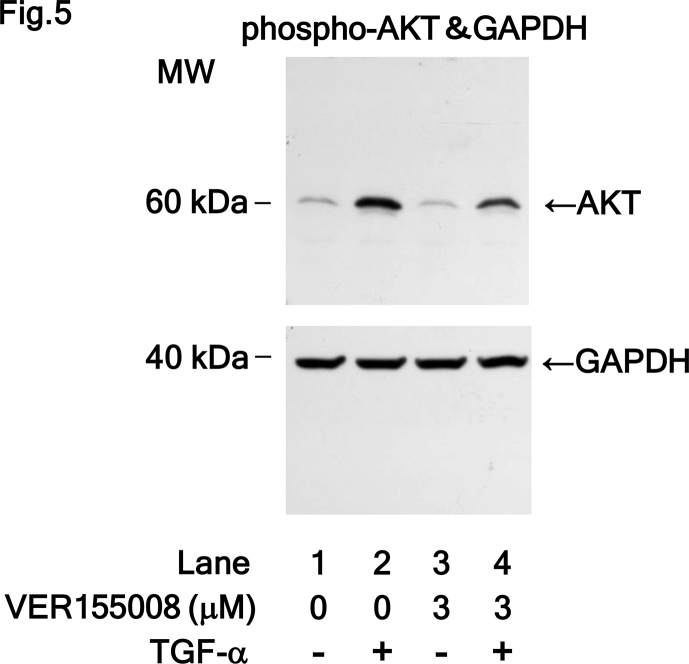


**S3:**
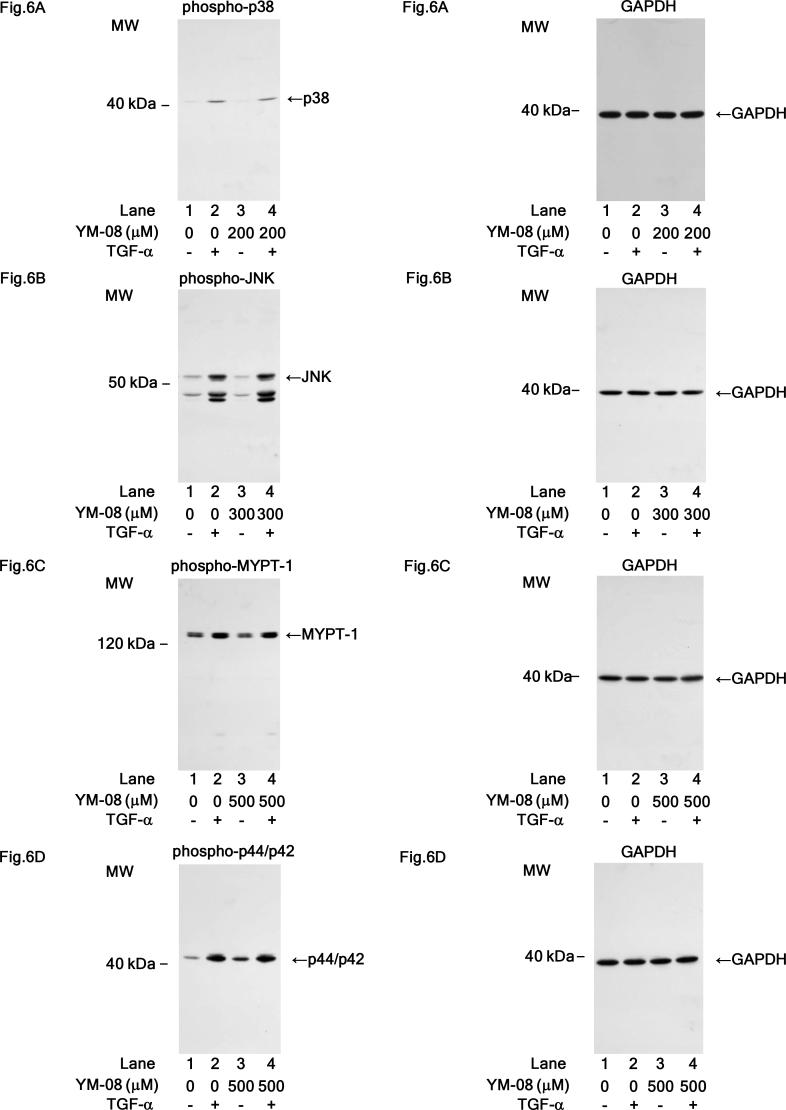


**S4:**